# Translation, cultural adaptation and validation of the Chinese version of the Carer Support Needs Assessment Tool for family caregivers of cancer patients receiving home-based hospice care

**DOI:** 10.1186/s12904-021-00766-7

**Published:** 2021-05-19

**Authors:** Sijia Zhou, Qianqian Zhao, Huimin Weng, Ning Wang, Xia Wu, Xinxin Li, Lili Zhang

**Affiliations:** 1 Shenzhen People’s Hospital, The Second Clinical Medical College of Jinan University, Shenzhen, Guangdong China; 2 School of Nursing, Southern Medical University, 1023 Sha Tai South Road, Bai Yun District, Guangzhou, 510515 Guangdong Province China

**Keywords:** Family caregivers, Cancer, Support needs, Palliative care, Validation, Hospice care, Carer support needs assessment tool

## Abstract

**Background:**

Family caregivers need to be supported in caring for patients at the end of life, but practical tools to assess their support needs have been missing in China. So this study aimed to culturally adapt and validate the Carer Support Needs Assessment Tool (CSNAT).

**Methods:**

Cross-cultural adaptation of the original CSNAT for a Chinese setting was performed according to Brislin’s translation guidelines. A pilot study was conducted with 15 Chinese family caregivers of cancer patients receiving hospice home care and 5 medical staff. A cross-sectional survey of 205 family caregivers was conducted from December 2018 to May 2019 at a home-based hospice care institute in Shenzhen, China. The validation procedure comprised the establishment of (1) content validity by a group of six experts; (2) face validity by 15 family caregivers; (3) criterion validity by calculating Spearman’s correlations between the CSNAT and caregiving burden, caregiving preparedness and quality of life scales; (4) internal consistency using Cronbach’s alpha.

**Results:**

The CSNAT demonstrated good face validity and good content validity. CSNAT scores showed clear positive correlations with caregiving burden and negative correlations with preparedness for caregiving and quality of life. Internal consistency was high (Cronbach’s alpha = 0.899), although such reliability testing is not recommended for this tool.

**Conclusions:**

The Chinese version of the CSNAT is a valid tool that is appropriate for identifying needs of family caregivers of cancer patients in home-based hospice care.

## Background

Cancer is a major public health concern worldwide and its incidence and mortality rates have rapidly increased in recent years [1]. Cancer affects both patients and their caregivers, as family members, relatives and friends undertake important care work and emotion management [2]. Cancer caregivers often report higher levels of burden and distress than those caring for frail older people or individuals with other diseases such as diabetes [3]. This is especially the case when cancer patients reach the end of life, as most patients prefer to die at home [4]. Their family caregivers therefore face more duties and responsibilities such as physical care, symptom management, emotional support and daily housework [5]. Owing to this considerable burden, many caregivers have unmet needs. If their needs are not addressed, they may experience reduced quality of life and greater distress [6]. International guidelines recommend that caregiver needs should be assessed and a program for caregiver support should be developed [7]. Therefore, it is important to assess and address the support needs of family caregivers, thus alleviating their caregiving burden and improving the quality of life of the whole family.

Sklenarova et al. [2] have suggested that strong predictors of caregiver needs remain to be identified; therefore, the specific needs of caregivers may need to be assessed individually. Professionals can only provide tailored assistance to this important group of persons if they can identify their specific care requirements. Thus, a comprehensive, effective and practical assessment tool is needed to help achieve this goal. The literature shows an increasing interest in assessing cancer caregivers’ needs using various instruments [8, 9]. However, these instruments are just a score, no follow-up support. The Carer Support Needs Assessment Tool (CSNAT) is a brief evidence-based tool developed by Ewing and Grande to identify family caregivers’ support needs for end-of-life care research and practice [10, 11]. It contains 14 domains on two broad groupings: Support to enable the carer to provide care and Direct support for the carers themselves. Generation of the CSNAT domains was based on focus group discussions and interviews with 75 bereaved carers. The tool has been subject to rigorous validation testing and has demonstrated good face, content and criterion validity [12]. Additionally, Alvariza et al. [13] demonstrated that the Swedish version of the CSNAT showed sound psychometric properties. The feasibility and validity of the tool were examined in a recent German study [14]. It is highly recognized by family caregivers and professionals. For use in practice, the tool is integrated into a 5-stage carer centred process of assessment and support, the CSNAT Intervention, that enables carers to consider, identify and prioritise their support needs, and then discuss prioritised needs and what they think may help with a practitioner, to enable supportive input to be tailored to carers’ own needs and preferences. Aoun et al. [15] reported that the CSNAT intervention significantly reduced caregiver strain. In 2020, Dr. Chan, W in Hong Kong translated this tool into traditional Chinese and applied it to caregivers of elderly patients in the community [16]. It focused on investigating the willingness of caregivers to accept end-of-life care, but did not clarify its reliability and validity. The tool is a traditional Chinese version, which is not suitable for application under the cultural background of mainland China. To the best of our knowledge, there is no such tool in China, and there are no reports on the translation of the CSNAT into Chinese and its application in China.

The study aim was therefore to translate the CSNAT into Chinese, to develop a culturally adapted version of the CSNAT for a Chinese-speaking context and to test its psychometric properties in China. A validated Chinese version of the CSNAT would be a useful tool for clinical policy makers and practitioners to identify support needs. Supportive input for family caregivers of end-of-life cancer patients can then be based on family caregivers’ needs and preferences.

## Methods

### Procedure

The study involved three stages: 1) translation of the CSNAT into Chinese, 2) cultural adaptation of the tool through a pilot study and expert committee review and 3) evaluation of the psychometric properties of the Chinese version of the CSNAT.

## Translation of the CSNAT

After permission was obtained from the CSNAT developers to translate the original tool into Chinese, the translation was performed according to Brislin’s translation guidelines [17]. A forward translation was conducted independently by two bilingual translators and group discussions were used to reach a consensus on a combined version. Without reading the original CSNAT, two different bilingual experts independently performed a backward translation of the Chinese CSNAT into English to establish semantic equivalence. The two back-translations were then reviewed by the research group. Finally, all the translators participated in a proofreading test, and an initial Chinese version of the CSNAT was established.

## Cultural adaptation of the CSNAT

Considering different clinical practice and diverse cultural backgrounds in the East and West, appropriate changes were made through an expert panel approach. The Chinese version of the CSNAT was reviewed by a panel of experts comprising two oncologists, two palliative care specialists and two psychological experts. They made comments on the relevance and clarity of each item of the tool using the 4-point Likert scales correspondingly: 1 = not relevant, 2 = a little relevant, 3 = relevant, 4 = highly relevant and 1 = very unclear needs full revisions, 2 = unclear and needs a bit of revisions, 3 = clear but needs only minor revisions, 4 = very clear not needs to be revised. The CVI was calculated at item level (I-CVI), with value more than 0.78 as recommended [18]. They also provided comments and suggestions about the tool. Subsequently, we conducted structured interviews with 15 family caregivers of cancer patients receiving home-based palliative care and 5 medical staff in the hospice care institution to seek their opinions on the tool items. They were asked whether each question was too difficult to understand or to answer. We also sought their suggestions for more appropriate phrasing of the questions if necessary. On the completion of the above steps, the final Chinese version of the CSNAT was established.

## Psychometric analysis of the Chinese version of the CSNAT

### Participants and data collection

Using a convenience sampling method, a cross-sectional study was conducted from December 2018 to May 2019 at a hospice care institution in Shenzhen, Guangdong, China. Participants were the primary family caregivers of end-of-life cancer patients. End-of-life cancer is defined as the presence of advanced metastases or estimated life expectancy of less than 6 months. Inclusion criteria were as follows: (1) over 18 years of age; (2) able to read and write Mandarin Chinese; (3) lives with the patient; (4) willing to participate in the research. Exclusion criteria were as follows: (1) having mental illness; (2) having an employment relationship with the patients. Researchers and participants conducted face-to-face assessments of support needs, and caregivers filled out additional questionnaires when they came to register at the hospice institute or when medical staff visited the patient’s home. Before the study was conducted, participants were given verbal and written explanations of the purpose and design of the study. It was emphasized that their participation was voluntary, that they could withdraw from the study at any time and that their responses would be anonymous and confidential. After this procedure, caregivers who agreed to participate provided the researcher with their written informed consent.

### Instruments

#### General information questionnaire

Sociodemographic and clinical data were collected using a self-report General Information Questionnaire. The questionnaire contained items on gender, age, relationship with patients, religious beliefs, type of residential area, occupational status, educational level, marital status, economic status and number of caregivers looking after the patient. The questionnaire also recorded the following patient clinical data: age, gender, type of cancer, metastasis, awareness of the disease, and whether patients had recently experienced pain, nausea, vomiting or difficulty breathing.

#### Carer support needs assessment tool

The 14 CSNAT items assess physical, practical, social, financial, psychological and spiritual support needs of cancer patient family caregivers during end-of-life care. Each domain was scored from 0 to 3: a score of 0 indicates ‘no need’; 1 indicates ‘a little more’; 2 indicates ‘quite a bit more’; and 3 indicates ‘very much more.’ Higher scores represent higher levels of need for help, although it should be noted that for carers themselves these gradations may not be that meaningful, and what matters is whether they need more support or not [13].

#### Preparedness for caregiving scale (PCS)

The PCS was originally developed by Archbold et al. [19] in the USA for use among family caregivers of frail older persons living at home, but has been used to assess caregivers’ preparedness to provide palliative care [20]. The PCS was translated into Chinese in 2016. It consists of eight items; each item is rated on a five-point Likert scale ranging from ‘not at all prepared’ (0) to ‘very well prepared’ (4). The total score ranges from 0 to 32; higher scores indicate more preparedness. Cronbach’s alpha for the PCS was 0.81 in the present study.

#### Terminal Cancer care load scale

The Chinese version of the Cancer Care Load Scale was validated by Zhang et al. [21] based on a survey of 186 advanced cancer family members. The scale is mainly used to measure the burden of family members of end-of-life cancer patients. It comprises 17 items measuring physical and mental burden, life burden, economic burden and spiritual burden. Each item is rated on a five-point Likert-type scale, ranging from ‘not at all’ (1) to ‘always’ (4). The total score ranges from 17 to 85; higher scores indicate a higher level of caregiving burden. In this study, Cronbach’s alpha for this scale was 0.93.

#### Item short-form health survey (SF-12)

The SF-12 was originally developed from the SF-36, which was widely used to assess self-reported health-related quality of life in various populations. In the survey of Chinese community elderly people by Shou J et al., the Cronbach’s α value of SF-12 was 0.910 [22]. The SF-12 consists of 12 items on eight dimensions. The scale includes two summary scores: a physical component summary score and a mental component summary score. Higher scores indicate better quality of life.

#### Karnofsky performance status scale (KPS)

The KPS was developed to measure patients’ functional status. Possible scores range from 0 to 100 with a standard interval of 10. Higher KPS scores indicate better function [23]. KPS was performed by the doctors or nurses at the time of the first home visit.

### Statistical analysis

The study database was established by two researchers using EpiData 3.1. Statistical analysis was performed using SPSS version 22.0. Descriptive statistics were used to describe participant personal characteristics and family caregiver needs. Content validity was assessed by six experts using a four-point rating scale: 1 = not relevant, 2 = somewhat relevant, 3 = quite relevant, 4 = highly relevant. The scale content validity index (S-CVI) was calculated as an mean of the item content validity index (I-CVI) for all items on the CSNAT. The I-CVI was calculated as the number of experts giving a rating of 3 or 4, divided by the number of experts. Content validity indices were rated as acceptable when I-CVI and S-CVI/Ave were respectively at least 0.78 and 0.90 [24]. Face validity was evaluated by 15 family caregivers and 5 medical staff. The test of criterion validity here assesses to what extent the tool relates to measures of constructs to which it theoretically should be related. To evaluate criterion validity, Spearman’s rank correlation was used to investigate the relationships between CSNAT items and preparedness for caregiving, caregiving burden and caregiver quality of life. We hypothesised that greater need for support would be associated with higher caregiving burden and worse preparedness for caregiving, quality of life and patient physical function. Internal consistency was calculated using Cronbach’s alpha coefficient to determine the reliability of the tool, although it should be noted that such reliability testing is not normally recommended for this tool [13].

### Ethical considerations

Ethical approval was obtained from the ethics committee of the Shenzhen People’s Hospital (approval no. LL-KY-201910) and permission to conduct the study was obtained from the Palliative Care Institute. All participants were notified of the research purpose and procedures and that they had the right to choose to participate or withdraw from the study at any time without penalty. All participants provided written informed consent and data confidentiality was guaranteed.

## Results

### Participant characteristics

A total of 218 family caregivers were invited to take part in the study. Five refused to participate because of high distress levels or inadequate time. Thirteen invalid questionnaires were excluded. Data for the remaining 205 caregivers were included in the analysis. The mean age of participants was 46.43 years (standard deviation = 11.33);53.2% were women and 46.8% were men. Participant demographic characteristics are shown in Table 1.

**Table 1:** Caregiver characteristics and patient clinical information (*n* = 205)

Variables	n	(%)
Caregiver		
Gender		
Male	96	46.8
Female	109	53.2
Relationship to the patient		
Spouse/partner	75	36.6
Son/daughter	108	52.7
Brother/sister	4	2.0
Friends	2	1.0
Daughter-in-law or son-in-law	16	7.8
Age (years), mean (46.43) SD (11.33)		
18–40	68	33.2
41–60	111	54.1
> 60	26	12.7
Type of residential area		
City	156	76.1
County	9	4.4
Town	26	12,7
Village	14	6.8
Marital status		
Unmarried	20	9.8
Married	172	83.9
Widowed	1	0.5
Divorced	12	5.9
Occupational status		
Employed	106	51.7
Unemployed	42	20.5
Sick leave	18	8.8
Retired	39	19
Family monthly income (RMB)		
High income	42	20.5
Upper middle income	65	31.7
Medium income	63	30.7
Low income	25	12.2
Extremely low income	10	4.9
Educational level (years)		
Primary school(1–6)	13	6.3
Junior high school(7–10)	55	26.8
Senior high school(11–14)	53	25.9
College level or above(> 15)	84	41.0
Religious beliefs		
No	162	79.0
Yes	43	21.0
Number of caregivers looking after the patient		
one	63	30.7
two	100	48.8
three or more	42	20.5
Patient		
Gender		
Male	120	46.8
Female	85	53.2
Age (years), mean (63.6) SD (13.57)		
18–40	8	3.9
41–60	75	36.6
61–80	97	47.3
> 80	25	12.2
Type of cancer		
Lung cancer	60	29.3
Colorectal cancer	41	20.0
Pancreatic cancer	20	9.8
Hepatic cancer	16	7.8
Reproductive system cancer	14	6.8
Gastric cancer	13	6.3
Breast cancer	12	5.9
Head and neck cancer	13	6.3
Esophageal cancer	5	2.4
Others	11	5.4
Metastasis		
Yes	204	99.5
No	1	0.5
Whether patients with unknown diagnosis		
Fully understand the condition	107	52.2
Know the diagnosis but do not know the prognosis of the disease	41	20.0
Not knowing the diagnosis at all	57	27.8
KPS score		
20	18	8.8
30	22	10.7
40	90	43.9
50	54	26,3
60	15	7.3
70	6	2.9

*SD* standard deviation, *KPS* Karnofsky performance status

**Fig. 1:**
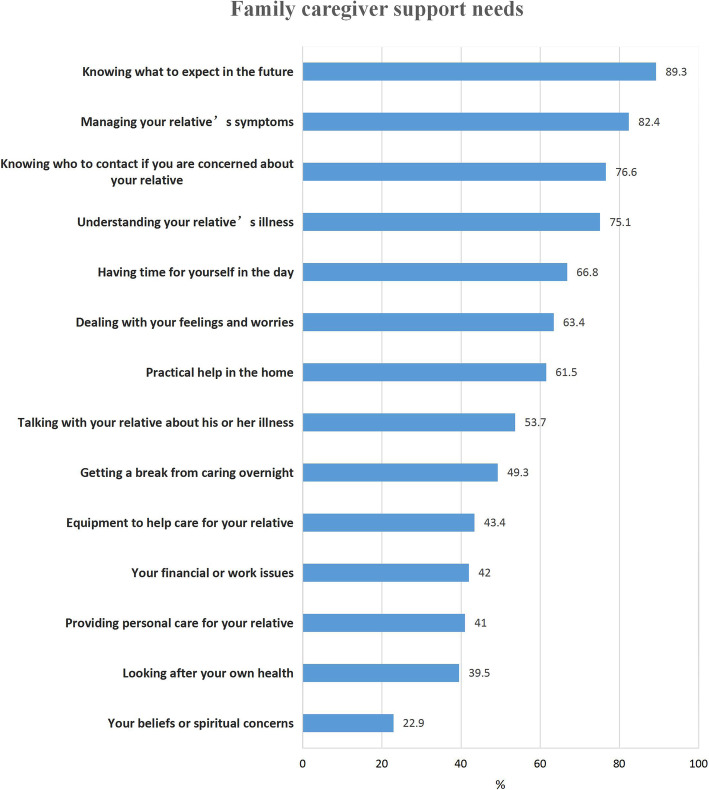
Family caregivers’ support needs as assessed by the CSNAT domains

A total of 89.3% family caregivers reported that they needed more support regarding knowing what to expect in the future and 82.4% caregivers reported that they needed more support with managing their relative’ s symptoms. More than 60% of caregivers reported that they wanted more support regarding knowing who to contact, understanding the illness, having time in the day for themselves, dealing with their feelings and worries, and obtaining practical help in the home. Only 39.5% needed support with their own health, and 22.9% needed support regarding their beliefs or spiritual concerns (Fig. 1).

### Content validity

The responses of the six experts produced item-level CVIs ranging from 0.83 to 1; the scale-level CVI was 0.98, indicating that the CSNAT had good content validity. The experts felt that all main issues were covered and they also offered specific feedback on wording and presentation. For example, in relation to item 9 (equipment to help care for your relative), one expert suggested adding information about the equipment (e.g., a wheelchair or oxygen machine). However, after consideration of the original authors’ purpose in developing the domain and further discussion with the expert group, this suggestion was not adopted. This domain does not refer solely to the type of equipment in use; rather, it reflects a broad domain that includes support needs relating to information about equipment, advice on what equipment may be useful, training to use equipment and reassurance that the equipment is being used correctly. Therefore, we did not add notes and explained the meaning of this domain to the experts. Another expert suggested that item 12 (practical help in the home) was not clear, as it did not specify the nature of the help (i.e., help from medical staff or other family members?). After consulting the author of the original CSNAT, we added an explanation of this item. This clarified that the item referred to help to manage housework or help around the home, and could encompass help from various sources, not just family members.

The analysis indicated that all the CSNAT items were used by the family caregivers in the study; none were redundant (Fig. 1), which is important. Although only a small percentage of family caregivers wanted more support with beliefs and spiritual concerns, this still represented 46 caregivers. A final optional question asked family caregivers if there were any additional issues they needed help with that were not addressed in the 14 items. Only 20 (5.8%) caregivers expressed an additional need that was not fully covered by the CSNAT, but these needs were strongly related to the existing CSNAT items: help providing emotional support to the patient, help to relieve the patient’s cancer pain, help to hire professional and attentive care workers, help with antitumor drugs and mediation methods, and economic help. It is possible that family caregivers just wanted to re-emphasize that they needed support in these areas. Thus, we concluded that the existing CSNAT items comprehensively covered family caregiver support needs.

### Face validity

Face validity was examined with family caregivers and palliative care staff. Fifteen family caregivers of terminal cancer patients were asked to participate in the pilot study; nine women and six men aged 25–62 years. The interview results indicated that the CSNAT was easy to complete. Participants felt that the tool gave them more opportunities to communicate with health care providers and express their emotions. However, 14 participants felt that the part of domain 4 that referred to legal issues was difficult to understand. This domain reflected the cultural background. In China, legal disputes between families are rare, as the culture emphasizes peace and harmony. Thus, after consulting experts, we removed this reference to legal issues. In addition, we also asked five health professionals in hospice care institute (two doctors, two nurses and one social worker) to give their opinion about the tool. They all felt that all domains were easy to understand and relevant to family caregivers. Thus, after consulting experts, we removed this reference to legal issues, as the need for this change was identified by carers to ensure cultural compatibility, and is therefore an acceptable change under CSNAT licence conditions.

### Criterion validity

Criterion validity here refers to whether the tool is related to measures of outcomes to which it theoretically should be related. Higher burden and lower quality of life are normally seen as outcomes of unmet needs and are related to preparedness [25, 26]. However, preparedness can be both a precursor and an outcome of support needs.

Table 2 shows the relationship between CSNAT items and scores on preparedness for caregiving, caregiver burden, caregiver quality of life and patients’ functional status. With the exception of insignificant correlations regarding the symptom management support need, all other CSNAT items had significant weak to moderate negative correlations with preparedness for caregiving (all *P* < 0.01). All items had significant weak to moderate negative correlations with family caregiver quality of life (all *P* < 0.05), but had significant weak to moderate positive correlations with caregiving burden (all *P* < 0.01). In addition, there were weak significant negative correlations between patients’ functional status and the following CSNAT items: knowing who to contact (*P* < 0.01), overnight and daytime breaks (*P* < 0.01), symptom management (*P* < 0.01), dealing with worries (*P* < 0.05), personal care (*P* < 0.05) and equipment needs (*P* < 0.05). These items were all related to the deterioration of the patient’s physical condition.

**Table 2:** Criterion validity: correlations between CSNAT domains and preparedness for caregiving, caregiver burden, caregiver quality of life and patient functional status (*n* = 205)

Carer Support Needs Assessment Tool Items	Preparedness for Caregiving	Caregiver Burden	Quality of Life	KPS
Understanding your relative’s illness	− 0.207**	0.181**	−0.154*	− 0.003
Having time for yourself in the day	−0.230**	0.279**	− 0.192**	− 0.189**
Managing your relative’s symptoms	−0.110	0.269**	−0.236**	− 0.205**
Your financial or work issues	−0.255**	0.251**	−0.161*	− 0.135
Providing personal care for your relative	−0.240**	0.263**	−0.291**	− 0.147*
Dealing with your feelings and worries	−0.340**	0.322**	−0.321**	− 0.155*
Knowing who to contact if you are concerned	−0.277**	0.333**	−0.238**	− 0.239**
Looking after your own health	−0.341**	0.339**	−0.393**	− 0.067
Equipment to help care for your relative	−0.243**	0.274**	−0.226**	− 0.159*
Your beliefs or spiritual concerns	−0.200**	0.199**	−0.187**	− 0.086
Talking with your relative about his or her illness	−0.355**	0.208**	−0.153*	− 0.126
Practical help in the home	−0.259**	0.413**	−0.321**	− 0.110
Knowing what to expect in the future	−0.188**	0.205**	−0.151*	− 0.108
Getting a break from caring overnight	−0.336**	0.352**	−0.330**	− 0.206**
The total of CSNAT	−0.395**	0.468**	−0.360**	− 0.222**

*CSNAT* Carer Support Needs Assessment Tool

Significant Spearman’s rank correlations, two-tailed test,*P-value < 0.05; **P-value < 0.01

### Reliability

Cronbach’s alpha for the whole tool was 0.899. The two CSNAT dimensions had Cronbach’s alphas of 0.808 and 0.831, respectively (Table 3). A high level of internal consistency was achieved. What needs to be pointed out that the paper by Alvariza et al. [13] and the earlier Ewing et al. [12] paper have noted that it is not really appropriate to test internal reliability of CSNAT, i.e. using Cronbach’s Alpha. This is because the tools seeks to capture the full range of caregivers’ support needs rather than a single underlying construct or constructs. So,it should be noted here that the results from such an analysis should be treated with caution.

**Table 3:** Internal consistency of the CSNAT (*n* = 205)

Dimensions	CSNAT items	Cronbach’s alpha coefficient
Support enabling the family caregiver to care	Understanding your relative’s illness	0.808
	Managing your relative’s symptoms	
	Providing personal care for your relative	
	Knowing who to contact if you are concerned	
	Equipment to help care for your relative	
	Talking with your relative about his or her illness	
	Knowing what to expect in the future	
Support in relation to their own well-being	Having time for yourself in the day	0.831
	Your financial or work issues	
	Dealing with your feelings and worries	
	Looking after your own health	
	Your beliefs or spiritual concerns	
	Practical help in the home	
	Getting a break from caring overnight	

*CSNAT* Carer Support Needs Assessment Tool

## Discussion

The purpose of our study was to translate the CSNAT into Chinese, and assess this tool’s validity and reliability in hospice home care with 205 family caregivers of terminal cancer patients. The findings indicated that the Chinese version of the CSNAT has good content, face and criterion validity, similar to the original English version and the Swedish version of the tool [12, 13]. Our study provides preliminary evidence of the internal reliability of the Chinese version of the CSNAT. However, psychometric testing of the CSNAT should be conducted with and interpreted with caution. CSNAT is not a questionnaire, but a communication tool to open up discussions with healthcare practitioners about the carers’ support needs within different broad domains of need, to capture the multidimensional and individual nature of carers’ support needs. The CSNAT is therefore not aiming to measure a single, underlying factor or to arrive at a single score for ‘need’. Further, whilst its response categories may help indicate level of need, categories are at best on an ordinal rather than interval scale, and may not be that meaningful to carers [13]. Tests of internal reliability, for instance, should therefore be interpreted with caution although they can give some indication of the tool’s characteristics.

The content validity of the Chinese CSNAT was reviewed by an expert panel and was based on expert ratings of item relevance. The results showed that all items on the Chinese version of CSNAT were considered appropriate and relevant. Both item-level and scale-level CVIs showed that the CSNAT has satisfactory content validity. Although it can provide some valuable insights, the content validity that only involves 6 people and only experts, with no carer involvement, is necessarily somewhat limited,

Face validity is very important, as it determines whether the tool can be used in practice. Qualitative feedback on the acceptability of the tool and its items was obtained from family caregivers and medical staff. To adapt the tool to the Chinese cultural background, we deleted reference to legal issues in domain 4. Such cultural adaptation of terms or situations that do not directly translate is permissible under the CSNAT licence, although amendments, additions or subtractions on any other basis would not be. The reason for deleting legal issues is that China is a society that emphasizes family ethics, and family relationships are important. In the face of death, legal disputes generally do not arise between family members and parents. This is considered unfilial. The response rate was high, nearly 97% of family caregivers participated in our research and interviews. Participants reported that the tool was brief, comprehensive and helpful. The survey made them feel that the medical staff cared about them and were allowing them the opportunity to express their needs and tell their stories.

The relationship between CSNAT items and caregiving burden, caregiving preparedness and quality of life scales indicated good criterion validity. There was also some correlation between CSNAT items and patient functional status; family caregivers require more support if patient physical condition is poor. These findings indicate that the tool has reasonable criterion validity.

Internal consistency reflects the interrelatedness of items on a scale and is a measure of whether all items assess the same construct. According to reliability testing CSNAT domains demonstrated a high level of internal consistency; Cronbach’s alpha was 0.899 for the whole tool and at least 0.808 for both groupings of support needs (Support to enable carer provide care and Direct support for carers themselves). Although such reliability testing is not directly applicable to CSNAT, it can give insights into some of its characteristics.

The findings indicated that as a tool, the CSNAT can help identify broad areas of need where carers require more support and open up conversations about what supportive input may help, to enable effective assessment of support needs of family carers in a hospice home care setting. The family caregivers in this study had similar support needs. The most frequent support need was associated with knowing what to expect in the future regarding patient care, a finding similar to that reported in a UK study [12]. The second most frequent support need was patient symptom management, especially pain management. One systematic review showed that pain management is the most obvious problem faced by family caregivers in end-of-life caregiving [27]. The least-mentioned support need was spiritual support, which reflects spiritual needs are not widely known at present. And compared with the suffering of loved ones, spiritual needs are not so important. Family caregivers generally expressed more need for support in caring for patients than for support for themselves.

In conclusion, the CSNAT is a valid and reliable tool that can be used in hospice care settings in China to identify supportive care needs of family caregivers of cancer patients.

## Limitations

There are several study limitations. First, the convenience sampling method may have caused sampling bias. Second, the data collection was only conducted in one hospice care institute, so the results may not be generalizable to other institutions.

## Conclusions

We translated and validated a Chinese (Mandarin) version of the CSNAT from the original English version. Our findings show that the CSNAT is suitable for assessing the support needs of family caregivers in home-based hospice care, and that the tool has good validity and reliability. The advantages of the CSNAT are its short format, ease of use in practice and comprehensive content. Our findings suggest that the CSNAT is a useful tool for healthcare providers to assess the support needs of family caregivers of end-of-life cancer patients in home-based hospice care in China.

## Data Availability

Due to the nature of this research, participants of this study did not agree for their data to be shared publicly, so supporting data is not available.

## Abbreviations

CSNAT: Carer Support Needs Assessment Tool
SF-12: item Short-Form Health Survey
PCS: Preparedness for Caregiving Scale
KPS: Karnofsky Performance Status Scale
